# Green Formulation of Spironolactone Loaded Chitosan-Coated Nano Lipid Carrier for Treatment of Acne Vulgaris: A Randomized Double-Blind Clinical Trial

**DOI:** 10.34172/apb.2024.011

**Published:** 2023-09-23

**Authors:** Majid Saeedi, Katayoun Morteza-Semnani, Jafar Akbari, Zohreh Hajheydari, Amin Goodarzi, Seyyed Sohrab Rostamkalaei, Seyyed Mohammad Hassan Hashemi, Seyyed Mobin Rahimnia

**Affiliations:** ^1^Pharmaceutical Sciences Research Center, Haemoglobinopathy Institute, Mazandaran University of Medical Sciences, Sari, Iran.; ^2^Department of Pharmaceutics, Faculty of Pharmacy, Mazandaran University of Medical Sciences, Sari, Iran.; ^3^Department of Medicinal Chemistry, Faculty of Pharmacy, Mazandaran University of Medical Sciences, Sari, Iran.; ^4^Department of Dermatology, Boo Ali Sina (Avicenna) Hospital, Faculty of Medicine, Mazandaran University of Medical Sciences, Sari, Iran.; ^5^Student Research Committee, Faculty of Pharmacy, Mazandaran University of Medical Sciences, Sari, Iran.; ^6^Department of Pharmaceutics, Faculty of Pharmacy, Ayatollah Amoli Branch, Islamic Azad University, Amol, Iran.; ^7^Department of Pharmaceutics, Faculty of Pharmacy, Hormozgan University of Medical Sciences, Bandar Abbas, Iran.

**Keywords:** Spironolactone, Acne vulgaris, Green formulation, Nano lipid carrier

## Abstract

**Purpose:**

Spironolactone (SPN), which is classified as an anti-androgen, has demonstrated efficacy in treating acne. This study aimed to utilize ultrasonication to create a chitosan-coated nano lipid carrier (NLC) for enhancing the delivery of SPN to the skin and treating acne.

**Methods:**

Various hydrophilic-lipophilic balance (HLB) values were investigated to optimize the SPN-NLCs. Photon correlation spectroscopy, attenuated total reflectance-Fourier transform infrared spectroscopy (ATR-FTIR), transmission electron microscopy (TEM), and differential scanning calorimetry (DSC) were employed to characterize the solid state of SPN in nanoparticle form. Additionally, the optimized formulation was used in a double-blind, randomized clinical trial.

**Results:**

Reducing the HLB of the surfactant mixtures resulted in a reduction in the size of SPNNLCs. The formula with the smallest particle diameter (238.4±0.74 nm) and the lowest HLB value (9.65) exhibited the highest encapsulation efficiency (EE) of 79.88±1.807%. Coating the optimized SPN-NLC with chitosan increased the diameter, polydispersity index (PDI), zeta potential (ZP), and EE. In vitro skin absorption studies demonstrated sustained release profiles for chitosan-coated SPN-NLC. In the double-blind trial, a gel containing chitosan-coated SPN-NLC effectively treated mild to moderate acne vulgaris, leading to improved healing and reduced lesion count after 8 weeks of therapy compared to the placebo. It successfully addressed both non-inflammatory and inflammatory lesions without adverse effects on the skin.

**Conclusion:**

The findings indicate that chitosan-coated SPN-NLCs have the potential as nanoparticles for targeted SPN delivery to the skin, offering novel options for the treatment of acne vulgaris.

## Introduction

 Acne vulgaris is a common inflammatory disorder of the pilosebaceous glands, making it one of the most prevalent dermatological conditions worldwide.^1^ Acne can persist throughout the adolescent years and negatively impact self-esteem. While there is no definitive cure for acne, many patients can discover a treatment regimen that effectively reduces the occurrence of lesions.^2^ Oral administration of various medications, such as oral contraceptives, certain antibiotics, and isotretinoin, is frequently prescribed to address chronic acne. However, there is a lack of robust evidence regarding the evaluation of the effectiveness of systemic acne treatments.^1^ Recent research indicates that for over 20 years, SPN has been used in tablet dosage form for the treatment of cutaneous diseases, specifically acne vulgaris, due to its proven efficacy.^3^ Clinical studies have demonstrated the anti-acne effects of spironolactone (SPN), and the outcomes of the treatment have been consistently positive.^4^

 Research has shown that daily oral administration of SPN at a dosage of 200mg has proven to be an effective alternative treatment for women with acne vulgaris.^5^ The main limitation in utilizing SPN has been its low solubility in aqueous solutions at physiological pH, which hampers its oral administration. Moreover, SPN exhibits poor absorption from the gastrointestinal system, and its therapeutic efficacy is limited due to variable oral bioavailability and potential endocrine-related adverse effects.^6^ Therefore, finding alternative routes of administration for SPN other than oral is crucial. Topical delivery of SPN is considered an ideal and accessible approach due to its various advantages observed in previous studies.^7^ Therefore, the extent of SPN permeation into the skin largely depends on the physicochemical properties of the material and the formulation of the vehicle used for topical delivery.^8^ With a molecular weight of 416.57 Da, a melting point of 212 °C, and a log P value of 2.78, SPN is considered an appropriate choice for topical administration. These properties contribute to SPN penetration and absorption into the skin.^9^ In certain situations, the penetration of organic chemicals through the skin may not be desirable. Therefore, in cases where systemic absorption is not necessary and increased absorption could lead to heightened adverse effects, some efforts need to minimize epidermal penetration.^10^

 Nanostructured lipid carriers (NLCs) are a relatively new type of colloidal drug delivery system that combines liquid and solid lipids. They offer improved drug encapsulation efficiency and release characteristics compared to traditional lipid-based carriers. These nanoparticles are composed of lipids that are non-irritating and non-toxic, making them well-tolerated for application on inflamed or injured skin.^11^ The small size of NLCs allows for direct interaction with the stratum corneum, the outer layer of the skin, thereby enhancing the penetration of encapsulated substances into the deeper layers of the skin, such as the dermis. This property facilitates the efficient delivery of therapeutic agents to the desired site of action.^7^ The utilization of this nanovehicle has played a crucial role in improving the dermal absorption of various pharmaceutical substances. Some of these substances are cyproterone acetate, isotretinoin, tretinoin, and adapalene. The use of the nanovehicle has demonstrated enhanced delivery and efficacy of these substances in dermatological applications.^12-14^ The positive outcomes observed in recent clinical trials using a topical preparation of SPN NLCs, which provides further evidence for the potential use of these nanovehicles in localized delivery of SPN. These trials demonstrated favorable results in acne patients, with no reported systemic hormonal alterations. This suggests that utilizing nanovehicles for localized SPN delivery could be a promising approach for acne treatment.^7,15,16^ Shamma and Aburahma conducted a study that effectively demonstrated the presence of SPN in scalp hair follicles. They also showed that SPN-encapsulated NLCs could reduce androgen synthesis within sebaceous glands and block the androgen binding site in cutaneous papillae. This research suggests that utilizing SPN-encapsulated NLCs for follicular delivery of therapeutic agents could be an effective treatment for alopecia.^17^ The effectiveness of 5% SPN gel in acne treatment was demonstrated by a significant reduction in overall lesion numbers and acne incidence score. However, the efficacy in treating non-inflammatory lesions (comedones) was found to be higher than inflammatory lesions (papules and pustules). This difference is attributed to the limited ability of SPN to penetrate specific microenvironments within inflammatory acne lesions.^16^

 Despite there have been a few investigations on the usage of NLC to enhance SPN cutaneous delivery, none of the researchers have examined the influence of dual surfactants and their hydrophilic-lipophilic balance (HLB) number on the production of SPN-encapsulated NLC and local delivery. SPN was selected as a lipophilic pharmaceutical substance for encapsulating into NLC through an ultrasonication process. Then the optimum SPN-NLC was coated with chitosan and applied on the skin for additional research. Furthermore, the effect of the dermal application of the chitosan-coated SPN-NLC gel in patients with acne vulgaris was studied in a double-blind, randomized clinical trial.

## Materials and Methods

###  Materials

 SPN was provided by Behdashtkar Co. (Tehran, Iran). Palmitic acid, Oleic acid, Tween 80, Span 80, acid acetic, propylparaben, methylparaben, and triethanolamine were obtained from Merck Co. (Germany). Chitosan low molecular weight was purchased from SIGMA (Germany). Carbopol 941 was provided by BF Goodrich (Cleveland, Ohio, 6 USA).

###  SPN-NLC preparation

 To prepare SPN-NLC, an ultrasonication method was employed.^18^ A magnetic heater-stirrer was used to melt a combination of palmitic acid, Span 80, oleic acid, and SPN at 95 °C. The aqueous phase, including Tween 80 and water, was heated to 75-80 °C. The warmed aqueous phase was then mixed with the lipids and SPN combination to form a pre-NLC using a magnetic stirrer. The resulting mixture was sonicated at 50% amplitude for 7.5 minutes and immediately placed in an ice bath while being agitated at 300 rpm. More details about the formulation can be found in Table 1.

**Table 1:** Formulations of SPN nanoparticles and physicochemical data (mean ± SD) (n = 3)

**Code**	**SPN (g)**	**PA (g)**	**OA (g)**	**Span 80 (g)**	**Tween 80 (g)**	**Water up to 80 mL**	**Chitosan (mg)**	**HLB**	**Particle size (nm)**	**PDI **	**Zeta potential (mv)**	**EE (%)**
F1	1.8	2	0.5	0	8	77.7	-	15	538.3 ± 12.34	0.542 ± 0.006	-4.3 ± 0.355	56.31 ± 0.656
F2	1.8	2	0.5	1	7	77.7	-	13.6	410.7 ± 5.40	0.517 ± 0.005	-3.7 ± 0.141	56.94 ± 0.516
F3	1.8	2	0.5	2	6	77.7	-	12.33	303.2 ± 4.25	0.441 ± 0.005	-3.1 ± 0.355	60.45 ± 0.858
F4	1.8	2	0.5	3	5	77.7	-	10.99	271.5 ± 6.20	0.389 ± 0.006	-3.0 ± 0.535	67.73 ± 1.302
F5	1.8	2	0.5	4	4	77.7	-	9.65	238.4 ± 0.74	0.397 ± 0.005	-2.8 ± 0.454	79.88 ± 1.807
F6 (chitosan-coated F5)	1.8	2	0.5	4	4	77.7	100	9.65	408.8 ± 2.37	0.505 ± 0.005	+ 7.3 ± 0.368	81.16 ± 0.565

SPN, spironolactone; PA, palmitic acid; OA, oleic acid; PI, polydispersity index; EE, encapsulation efficiency.

 For the production of chitosan-coated SPN-NLC, 100 mg of low molecular weight chitosan was dissolved in 100 ml of 1% glacial acetic acid in water and Tween 80. This chitosan solution was added dropwise to 80 ml of the raw NLC while stirring at 400 rpm. Stirring was continued for 3 hours after the addition of the chitosan solution. This process was carried out using the optimum formulation (F5).

###  Characterization of preparations

 The mean diameter, polydispersity index (PDI), and zeta potential (ZP) of the NLC were determined using a Zetasizer Nano-ZS device equipped with dynamic light scattering (DLS) technology (Malvern Instruments Ltd., UK).^3^ The interaction between the components was assessed using a Cary 630 FTIR spectrophotometer with a diamond attenuated total reflectance (ATR) attachment (Agilent Technologies Inc., CA, USA). Infrared spectra were obtained in the range of 4000-400 cm^-1^ with a resolution of 2 cm^-1^.^19^ Differential scanning calorimetry (DSC) measurements were carried out using a pyris6 instrument (PerkinElmer, Norwalk, USA). The morphological characteristics of the nanoparticles were examined using an EM 208S transmission electron microscope (TEM) (Philips, Netherlands).

###  Encapsulation efficiency (EE%)

 To determine the EE% of SPN in the NLCs, the SPN-NLCs were subjected to centrifugation for 90 minutes at 27,000 rpm using a HERMLE Z36HK centrifuge (Germany). The resulting supernatant was filtered through a 0.22 μm pore size filter, and the amount of free drug in the supernatant was quantified using a Knauer XDB-C18 column (5 μm, 4.6 × 250 mm) at a wavelength of 238 nm. The mobile phase used was a mixture of 70% acetonitrile and 30% ultrapure water (v/v), delivered at a flow rate of 0.8 ml/min. The EE was calculated using equation 1.^3^


$$EE\%=\left(\frac{Winitial-Wfree}{Winitial}\right)\times 100$$

###  In vitro drug release

*In vitro* release test was conducted by putting the samples in immersion cells covered by a cellulose acetate membrane with a molecular weight cut-off (MWCO) of 12 kDa and sealed with a stopper. These cells were then placed in modified USP dissolution apparatus II. To minimize the volume of dissolution media, the vessels were replaced with 250 mL beakers, and 70 mL of phosphate buffer solution with pH = 5.8 was used as the dissolution media in each beaker. At specific intervals (2, 4, 6, 8, and 24 hours), 5 mL of the dissolution medium was withdrawn and filtered through a 0.22 μm filter paper. The filtered samples were then analyzed using high-performance liquid chromatography (HPLC), and the amount of substance present at 238 nm was quantified (refer to section 2.4 for specific HPLC parameters). After each sample collection, 5 mL of phosphate buffer solution was added back to the dissolution media to maintain a constant volume.^3^

###  Gel preparation

 To prepare the plain gel, Carbopol 941 was dispersed in preserved water at a concentration of 8% w/v and left for 24 hours. The Carbopol solution was then neutralized by adding 300 mg of triethanolamine. To prepare the chitosan-coated SPN-NLC (1%) gel, 160 g of chitosan-coated SPN-NLC (containing 1.8 g of SPN) was mixed with 20 g of plain gel using a propeller homogenizer at 400 rpm. Similarly, to obtain the SPN-NLC (1%) gel, 160 g of SPN-NLC (containing 1.8 g of SPN) was mixed with 20 g of plain gel using a propeller homogenizer at 400 rpm. To create SPN 1 % simple gel, a specific quantity of SPN was dispersed in deionized water and combined with plain gel through the same mixing procedure.

###  In vitro skin absorption

 The animal studies conducted in this research were approved by the MAZUMS’ Ethics Review Board for Animal Research under registration code IR.MAZUMS.REC.1399.691. Male Wistar rats weighing between 120 and 150 g were used for the experiments. The rats were anesthetized with a combination of 87 mg/kg ketamine and 13 mg/kg xylazine. Their abdominal skin was then carefully cut using electric hand scissors. After 48 hours, the rats were euthanized by inhalation of chloroform, and the abdominal skin was surgically removed. The excised skin was thoroughly washed to remove any adhering fats and then treated with normal saline for 24 hours at 4 °C before the diffusion studies began. Franz cells were used for the experiments. The removed skin was placed between the donor and receiver compartments, with the dermis facing the receiver medium. The receiver compartment was filled with buffer phosphate, and the diffusion cells were maintained at a temperature of 32 ± 0.5 °C with agitation at 150 rpm. In the donor chamber, 5 grams of SPN-NLC gel containing 50 000 µg of SPN and 5 grams of chitosan-coated SPN-NLC gel with an equivalent amount of SPN in SPN-NLC gel were evenly applied to the shaved dorsal regions. The donor chamber was isolated from the environment. At predetermined intervals (2, 4, 6, 8, 10, and 24 hours), 5 mL of samples were collected from the receiver compartment, and an equal volume of fresh 5 mL buffer phosphate was added to maintain the volume. The amount of SPN in the collected samples was evaluated using HPLC at a wavelength of 238 nm.^3^

###  SPN skin retention assessments

 After the completion of the penetration experiment, the excised skin samples were removed from the diffusion cells. The skin samples were washed three times with deionized water to remove any residual formulation and then dried. The amount of SPN retained in the skin was determined. The washed skin samples were cut into smaller pieces using clippers and placed in a tube. They were then digested in water for 24 hours. After the digestion period, the samples were sonicated for 1 hour at room temperature in a bath sonicator. This step was performed to ensure the complete extraction of SPN from the skin. The supernatant obtained after sonication was separated from the skin debris and passed through a syringe filter with a pore size of 0.22 µm to remove any particulate matter. The filtered solution was then used to determine the amount of SPN using HPLC at a wavelength of 238 nm.^3^

###  Clinical trial design

 In the randomized controlled trial, specific criteria were used to select the volunteers. Those who had received systemic or local anti-acne medication within the past 3 months or during the study were excluded from participation. Pregnant volunteers, individuals planning to become pregnant, lactating volunteers, and individuals with cutaneous disorders that could interfere with the assessment of hyperpigmentation were also excluded. The study included 40 cases of mild to moderate acne. The severity of acne was assessed using the global acne grading system (GAGS rating), with scores ranging from 1 to 30. Participants within the age range of 8 to 65 years (22 ± 5.76) were eligible for inclusion in the study. These criteria were used to ensure that the study participants had a specific acne severity level and were not affected by factors that could potentially interfere with the study outcomes, such as previous medication usage or specific medical conditions.^20^

 After obtaining signed informed consent, individuals who expressed dissatisfaction with their previous acne treatments were enrolled in the double-blind clinical research investigation. Prior to the start of the trial, the Mazandaran University of Medical Sciences ethics board provided their approval with the registration code IRCT20120707010203N11.

 During the initial appointment, each patient completed a detailed questionnaire that gathered information about their demographics, acne history, and clinical background. Subsequently, the participants were randomly assigned to one of two therapy groups: Treatment group received chitosan-coated SPN-NLC gel + clindamycin 2% solution, while placebo group received chitosan-coated NLC gel + clindamycin 2% solution. Both the therapists and participants were unaware of the specific therapy assigned to each group, ensuring a double-blind study design. Participants were instructed to cleanse their faces with non-medicinal soaps in the morning and evening and ensure they were thoroughly cleaned and dried. Throughout the 8-week course of the study, each participant received two tubes of the prepared gel and was instructed to apply approximately 2 cm (around the size of a knuckle) of the gel to the affected area each morning and evening, gently massaging it for approximately 2 minutes. The gel was applied specifically to acne lesions and left on for 2-3 hours before being rinsed off. Non-medicated cosmetics were allowed during the research period.

 Participants were regularly questioned about their compliance with the treatment procedure and any adverse effects they experienced. Throughout the study, participants were prohibited from using any other medications or acne-related skincare products to ensure the efficacy and safety of the tested therapies.

###  Clinical evaluations

 Acne vulgaris, the most prevalent skin disorder, is characterized by the presence of comedones or intense inflammatory lesions. The development of comedones in acne-prone skin is attributed to the overproduction of the male hormone androgen and sebaceous glands.^21^ The two main types of noninflammatory lesions in acne are closed comedones (whiteheads) and ripen comedones (blackheads). If the contents of these lesions rupture, they can develop into inflammatory papules and pustules. Additionally, larger and more painful lesions like cysts and nodules can form. Acne papules are solid, inflamed bumps on the skin that typically have a cone-shaped appearance. Unlike pustules, they do not have a pus-filled tip. Conversely, pustules display a notable amount of pus, appearing red at the base and featuring a yellow or white center surrounded by small, inflamed round lesions.^22,23^

 At every clinical examination, the participants’ facial lesion numbers were evaluated for alterations (noninflammatory lesions: open and closed comedones; inflammatory lesions: papules and pustules) (0 and 8 Weeks). During each examination, the evaluation involved assessing the frequency, type, and distribution of acne lesions. The preceding two equations were utilized for the overall evaluation and to calculate the treatment’s efficiency.^16^

 Total Lesion Count (TLC) = comedones + papules + pustules

 Acne Severity Index (ASI) = papules + (2 pustules) + (comedones/4).

 The multi-test equipment was used to evaluate the inflammation, irritation, itching, and redness of the skin at every session (MC 900; Enviro derm, Gloucestershire, UK). From the base, the indications and complaints were assessed every two weeks.^7^

###  Statistic evaluation

 The data was analyzed using SPSS version 22.0, developed by IBM (USA). ANOVA was conducted to assess the identified parameters, followed by the Tukey test as the post hoc test. The student’s t-test was used to examine changes in skin-related factors. A significance level of *P* < 0.05 was considered statistically significant.

## Results and Discussion

###  Properties of the SPN-NLC preparation

 The NLC encapsulating SPN was prepared using the ultrasonication technique. Different ratios of binary mixes of two surfactants (Tween 80 and Span 80) were used to achieve various HLB levels and enhance the SPN-NLC formulation. The particle diameter of the nanoparticles was a key focus of this study. Table 1 presents the hydrodynamic diameter of the nanoparticles, while the PDI values in Table 1 represent the particle size distribution, indicating the uniformity or heterogeneity of the particle sizes. The PDI values range from 0 to 1, and a value greater than 0.6 indicates a broad distribution of particle diameters in the sample. A PDI value above 0.6 suggests a wide range of particle sizes within the specimen.^18^

Table 1 illustrates that reducing the HLB value of binary surfactant combinations from 15 (F1) to 9.6 (F5) resulted in a significant decrease in the diameter of SPN-NLC nanoparticles from 538.3 ± 12.342 to 238.4 ± 0.74 nm (*P*< 0.05). In order to achieve a formulation with a lower HLB, the amount of Span 80 (a surfactant with a minimum HLB value) needs to be increased, which facilitates the entrapment of more pharmaceutical substances into the nanoparticles. Optimal HLB values during the production process are expected to result in smaller emulsion droplets. As the HLB value of a dispersion approaches the required HLB of the lipids, particles with smaller diameters are generated, resulting in a more stable system.^24^ This could explain why F5 exhibited a smaller diameter with a lower PDI. The inclusion of binary surfactant combinations in the formulations can also improve preparation stability. By dispersing surfactants with higher HLB values in the aqueous phase and surfactants with lower HLB values in the oil phase, the use of minimum/maximum HLB surfactant blends ensures enhanced stability of the emulsion droplets.^25^ Achieving surfactant film stability at the interfaces between the reservoirs in each stage can be accomplished by distributing the surfactant with a lower HLB value in the lipid phase. This arrangement promotes the formation of a stable film, which helps maintain the integrity of the interfaces and enhances the overall stability of the system.^26^

 The significant decrease in interfacial tension between the lipid and aqueous phases, leading to improved homogenization of the lipid in the aqueous medium, could account for the smaller diameter of nanoparticles containing a high content of two surfactants with higher and lower HLB values. Consequently, the incorporation of these surfactants can contribute to the reduction in nanoparticle size.^3^ Elmowafy et al demonstrated that a formulation with a low HLB number can effectively reduce the diameter of hydrophobic pharmaceutical substances, such as atorvastatin, when entrapped in NLCs.^27^

 The PDI of the NLC formulation prepared using a binary combination of surfactants was 0.6, indicating an acceptable size distribution of the NLCs. The ZP of the various preparations was found to be negative, ranging from -4.300 ± 0.355 mV (F1) to -2.800 ± 0.454 mV (F5). This negative ZP can be attributed to the dipolarity of the ethoxy segments present in nonionic surfactants, which leads to the development of a negative electrical charge around the nanoparticles.^28^ When binary combinations of Span and Tween surfactants are used instead of a single surfactant, there is a higher probability of forming a rigid barrier around the nanoparticles, leading to the enhancement of their negative charge. This can be attributed to the increased presence of Span in the formulation, which results in fewer surfactants like Tween 80 binding to the nanoparticles. As a consequence, the ZP of the nanoparticles decreases.^29^ Due to the low critical micelle concentration of Tween 80 (approximately 0.015 mM), it is necessary to minimize the amount of Tween 80 subunits in the dispersion media. This is to ensure that the surfactant remains in its monomeric form and does not form micelles, which could have an impact on the stability and properties of the formulation.^30^ This indicates that instead of participating in micelle formation, surfactant subunits are more likely to adsorb onto the hydrophobic surface of lipids. Surfactants align themselves with their hydrophobic moiety in the lipid phase and their hydrophilic moiety in the aqueous phase at the interface of two immiscible fluids, such as water and lipids. Nonionic surfactants have been observed to fully cover the surfaces of nanoparticles, providing stability and preventing aggregation.^29^ Excessive amounts of the secondary surfactant (in this case, Span) can hinder the primary surfactant’s ability to provide an effective coating.^18^ The addition of a secondary surfactant, particularly in large quantities, may disrupt the surfactant film surrounding the nanoparticles, resulting in a decrease in the ZP. In this study, the ZP values were not considered a primary factor for selecting the optimal preparation. The NLCs were effectively protected by nonionic surfactants such as Tween 80 and Span 80, which provided steric stabilization, despite having a very low ZP, thereby ensuring greater stability.^18^

 The evaluation of EE% revealed that it ranged from 56.31 ± 0.656% (F1) to 79.88 ± 1.807% (F5) for the different preparations. The EE% can be influenced by the pharmaceutical’s miscibility and solubility in the lipid medium, as well as the polymorphism of the lipid phase.^30^ Formulations with lower HLB levels, such as the one with an HLB level of 9.65 and smaller particle sizes, exhibited the highest EE%. Previous research has indicated that formulations or lipids with lower HLB levels are more favorable for enhancing the solubility of hydrophobic substances like SPN. Consequently, NLC formulations with lower HLB levels can effectively entrap a greater amount of medication within the lipid matrix.

 The new approach for producing SPN-NLC offers advantages over previously disclosed techniques as it eliminates the need for an organic solvent. Furthermore, the impact of the Span: Tween ratio on SPN EE in NLC has not been investigated in prior studies. The prepared NLC formulation was incorporated into a gel base, allowing for the development of a pharmaceutical dosage form. The optimized SPN-NLC formulation achieved a particle diameter of 238.4 ± 0.7 nm, a PDI of 0.397 ± 0.005, a ZP of -2.8 ± 0.4 mV, and a drug encapsulation percentage of 79.88 ± 1.80%. Due to desirable and acceptable characteristics, including the smallest particle size and appropriate PDI, as well as having the highest SPN EE, the F5 formulation was selected as the optimum SPN-NLC and was employed for coating with chitosan.

Table 1 demonstrates the influence of chitosan on the average particle diameter of chitosan-coated SPN-NLCs. With a 2% emulsifier content, the average particle diameter of chitosan-coated SPN-NLCs increased from 238.4 ± 0.74 nm (F5) to 408.8 ± 2.37 nm. Due to the electrostatic interaction between the negatively charged surface of NLCs and the positive charge of chitosan, the surface charge of chitosan-coated SPN-NLCs was significantly altered from -2.8 ± 0.454 mV (F5) to + 7.3 ± 0.368 mV (*P*< 0.05). Additionally, the PDI value showed a significant change from 0.397 ± 0.005 (F5) to 0.505 ± 0.005. The presence of the positively charged chitosan coating resulted in an increase in the average particle diameter, PDI value, and ZP of chitosan-coated SPN-NLCs.^31^ Table 1 provides the encapsulation efficiency of SPN-encapsulated NLCs and chitosan-coated SPN-NLCs. It can be observed that chitosan-coated SPN-NLCs exhibited slightly higher encapsulation efficiency (81.16 ± 0.565%) compared to F5 SPN-NLCs (79.88 ± 1.807%), although the difference was not statistically significant (*P >*0.05). The increased EE% observed in chitosan-coated SPN-NLC, in comparison to uncoated SPN-NLC (F5), is likely a result of the larger particle size in the coated NLC system. The larger particles offer more space for encapsulating the bioactive materials within the core or matrix of the particles, leading to higher EE%. Additionally, the interaction between the positive charge of chitosan and the negative charge of uncoated SPN-NLC contributes to the higher EE% in the chitosan-coated SPN-NLC.^32,33^

###  TEM examination

**Figure 1:**
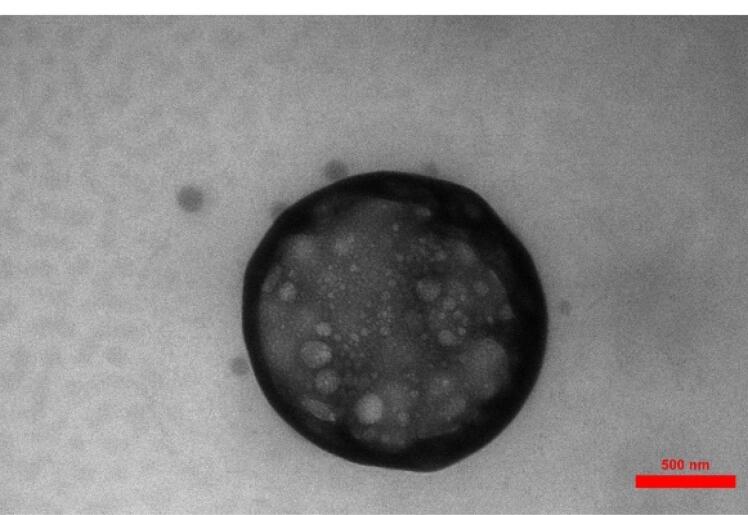
TEM pictures of F6 (chosen preparation)

 Formulation 6 (F6) was chosen as the optimal preparation to show nanoparticle microscopy images, as shown in Figure 1. Figure 1 depicts spherical chitosan-coated NLC particles, clearly exhibiting the presence of the chitosan coat surrounding the NLCs.

 The difference in size between the DLS and TEM results can be attributed to several factors: Measurement technique, Sample preparation, Instrument limitations, and Sample characteristics. The measurement technique is one of the most important factors that affect size variation. The authors discussed the reason for this difference in size between DLS and TEM results. DLS measures the hydrodynamic size of particles or molecules in a solution, while TEM directly images the size of individual particles using an electron beam. DLS provides an average size based on the Brownian motion of particles, whereas TEM provides direct visualization of individual particles.^26^

###  DSC investigation

**Figure 2:**
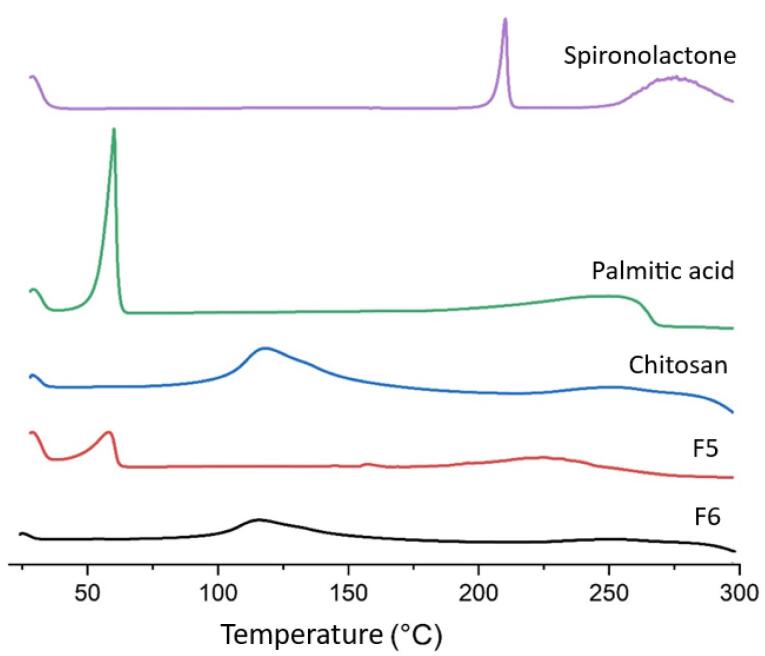
DSC graph of Spironolactone, Palmitic acid, Chitosan, F5 and F6

Figure 2 illustrates the DSC behavior of pure SPN, pure palmitic acid, chitosan, F5, and F6 (chitosan-coated SPN-NLC) powders. In the DSC graph of SPN and palmitic acid, a distinct sharp endothermic peak was observed at 212 °C and 60 °C, respectively, indicating their melting points. The DSC thermograms of chitosan in Figure 2 show a small endothermic peak which was probably related to the elimination of moisture content in range of 100 to 118 °C.^19,34^ According to the report, it was found that DSC does not typically give information about the glass transition temperatures or melting temperatures of polysaccharides. Additionally, our study revealed that no DSC peak was observed for chitosan, indicating that there will be no thermal changes for chitosan up to 300 °C.^35,36^ Figure 2 also demonstrates the DSC behavior of F5 preparation, where only one endothermic peak corresponding to the melting point of palmitic acid is observed, but the main endothermic peak of the drug has disappeared. This suggests that the drug may be in an amorphous phase within the preparation, potentially due to the solubilization of SPN in a polymeric matrix. In all the DSC graphs shown in Figure 2, the melting peaks of the drug and palmitic acid are absent in the chitosan-coated SPN-NLC formulation. This indicates that the coating process with chitosan has been successfully carried out, possibly resulting in the presence of amorphous SPN in the preparations. The DSC graph of F6 exhibits the melting peak of chitosan, albeit with lower intensity. This lower intensity can be attributed to the fact that the preparation contains a lower level of chitosan compared to pure chitosan in the DSC traces.

###  ATR-FTIR evaluation

**Figure 3:**
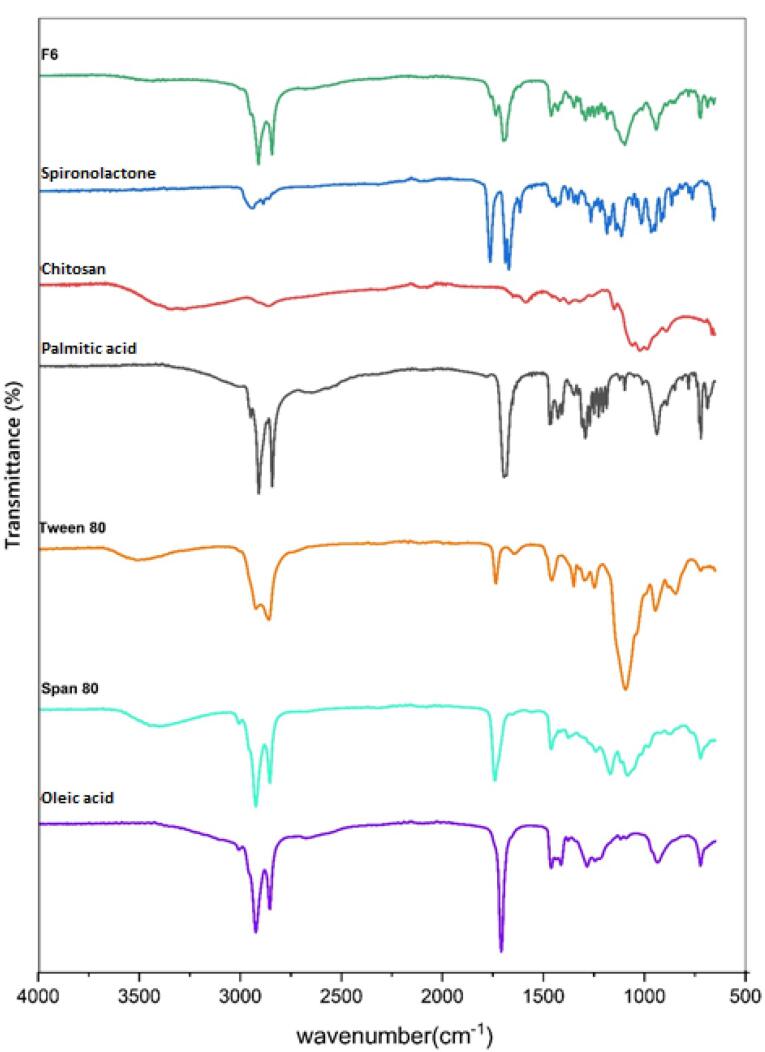
ATR-FTIR spectra of F6 formulation, Spironolactone, Chitosan, Palmitic acid, Tween 80, Span 80, and Oleic acid

 The possible chemical interaction of SPN with the other components of the formulation was analyzed by ATR-FTIR spectroscopy. The ATR-FTIR spectra of pure SPN, palmitic acid, oleic acid, Tween 80, Span 80, chitosan, and F6 formulation were reported in Figure 3. The *characteristic* peaks in the ATR-FTIR spectrum of SPN were identified at 2949 cm^−1^ & 2893 cm^−1^ (C-H stretching), 1765 cm^−1^ (C = O stretching of lactone), 1690 cm^−1^ (C = O stretching of thioacetyl group), 1673 cm^−1^ (C = O stretching of α, β-unsaturated ring), and 1618 cm^−1^ (C = C stretching).

 The ATR-FTIR spectrum of chitosan showed the main peaks at 3600-3100 cm^-1^ (N-H & O-H stretching), 2915 cm^-1^ & 2863 cm^-1^ (C-H stretching), and 1060 cm^-1^ & 1022 cm^-1^ (C-O stretching).

 The ATR-FTIR spectrum of palmitic acid indicated the peaks 3400-2400 cm^-1^ (O-H stretching of the carboxylic acid group), 2915 cm^−1^ & 2848 cm^−1^ (C-H stretching), and 1697 cm^−1^ (C = O stretching of the carboxylic acid group).

 The spectrum of Tween 80 displayed the peaks at 3502 cm^-1^ (O-H stretching), 2922 cm^-1^ (-CH_2_-asymmetric stretching), 2859 cm^-1^ (-CH_2_- symmetric stretching), and 1735 cm^-1^ (C = O stretching).

 The spectrum of Span 80 showed the peaks at 3401 cm^-1^ (O-H stretching), 2923 cm^-1^ (-CH_2_- asymmetric stretching), 2854 cm^-1^ (-CH_2_- symmetric stretching), and 1739 cm^-1^ (C = O stretching).

 The spectrum of oleic acid presented the peaks at 3400-2400 cm^-1^ (O-H stretching of the carboxylic acid group), 3007 cm^-1^ (C-H stretching of C = C-H), 2923 cm^-1^ (-CH_2_- asymmetric stretching), 2854 cm^-1^ (-CH_2_- symmetric stretching), 1708 cm^-1^ (C = O stretching of the carboxylic acid group), and 1284 cm^-1^ (C-O stretching of the carboxylic acid group).

 According to the ATR-FTIR findings, there was no chemical interaction between the SPN and other materials of the F6 preparation, since the characteristic peaks of SPN including C = O and C = C stretching peaks can be detected in the ATR-FTIR spectrum of the F6 formulation without shift.

###  Release of the drug in in vitro condition

**Figure 4:**
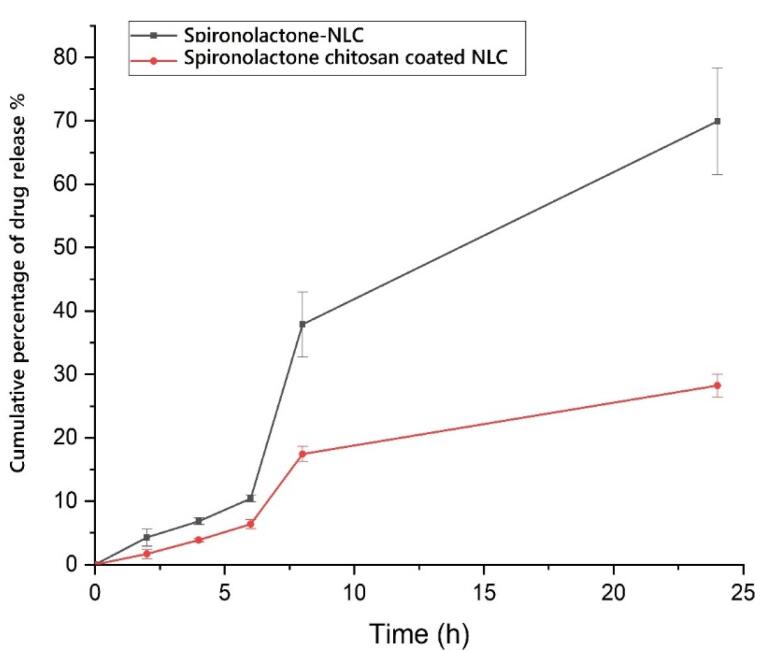
Cumulative percent of drug release from preparations

 Modulating the drug release from nanoparticles (NPs) has been found to have a direct impact on the bioavailability of pharmaceutical substances.^37^ In diffusion-controlled release studies, it was observed that chitosan-coated NLC exhibited a gradual release of SPN over time. During the initial 2 hours, only 1.673 ± 0.709 percent of SPN was released, followed by a sustained release profile reaching 28.244 ± 1.769 percent after 24 hours. This release pattern can be attributed to the regulated release pathways and diffusion of SPN from the lipid core matrix of the NLC and the chitosan coating into the dissolution medium. Notably, the release rate of SPN from chitosan-coated NLC was significantly slower compared to SPN-NLC (Figure 4) (*P*< 0.05). *In vitro* release experiments confirmed that the release of SPN from chitosan-coated NLC exhibited a longer and more sustained profile over 24 hours. These results indicate that the chitosan-coated NLC formulation provides a controlled release of SPN. The presence of SPN in the lipid matrix, enriched lipid core, and chitosan shell surrounding the NLC contribute to the extended-release rate of the pharmaceutical substance. The chitosan coating acts as a barrier, leading to a reduction in release rates and prolonging the path of the drug from the core of the nanoparticles to the external release medium.^38^ Likewise, the formulation of coumarin as chitosan-SLN resulted in improved efficacy and bioavailability, as well as enhanced release behavior of the preparations. The chitosan-SLN formulation exhibited prolonged release profiles, contributing to the sustained delivery of the drug. This prolonged release pattern enhances the therapeutic effectiveness of the drug and improves its bioavailability by maintaining a consistent concentration of the drug in the target site over an extended period.^39^

###  Skin absorption test

 Pharmaceutical substance penetration into and throughout the skin is a determining factor in assessing the suitability of preparation for transdermal (penetration throughout the skin) or dermal (penetration into the epidermis and dermis) applications. While rat skin has commonly been used in cutaneous penetration studies, it has been increasingly recognized that human skin provides more accurate permeability data compared to rat skin. Extensive research has shown that the use of human skin yields more reliable and relevant results in assessing cutaneous permeability. Therefore, the utilization of human skin in cutaneous penetration studies is preferred to obtain more accurate insights into the penetration behavior of pharmaceutical substances.^40^ Furthermore, human skin is less absorbent than that of rats.^41,42^ In order to assess the effectiveness of chitosan-coated SPN-NLC formulations compared to SPN-NLC, rat skin was employed as a model for cutaneous evaluation. The choice of rat skin as a template was made with the specific aim of studying and comparing the performance of the formulations under investigation.

**Figure 5:**
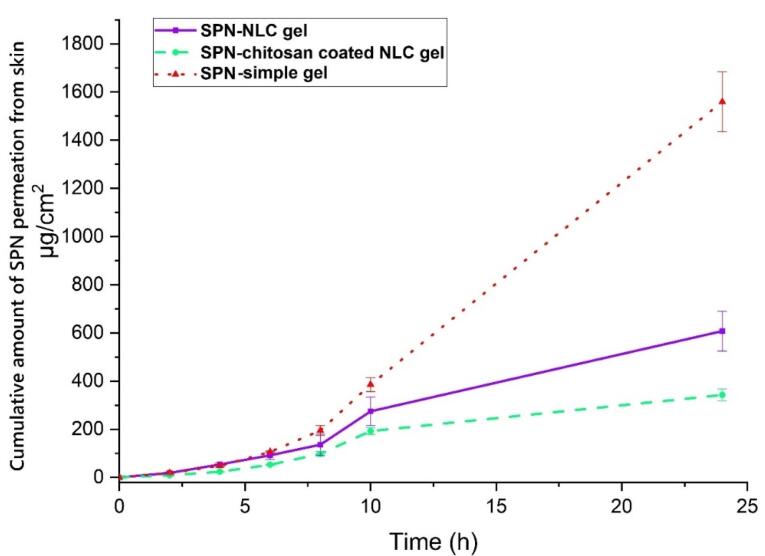
The total average extent of spironolactone permeated across rat epidermis (records are average and SD of three assessments, n = 3)

**Figure 6:**
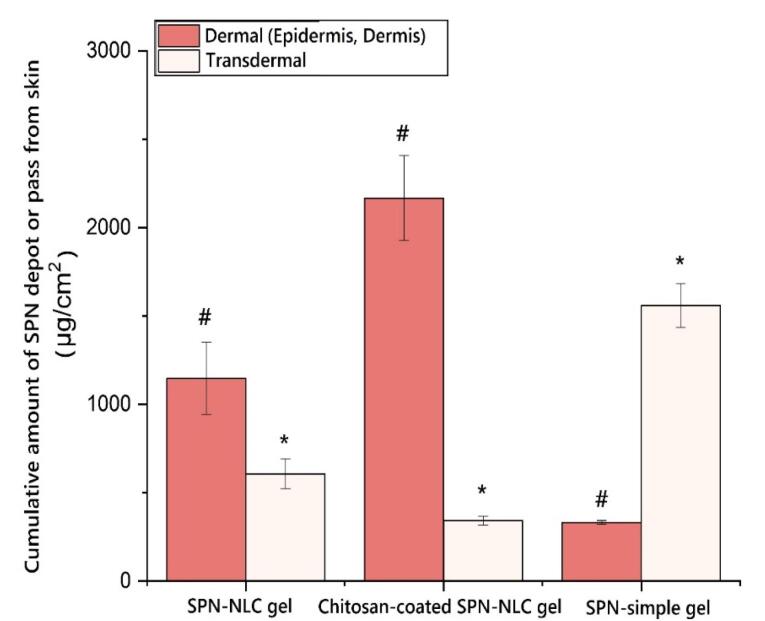
The extent of spironolactone penetrating the epidermis layers (local targeting). * *P*< 0.05 in comparison to the SPN-simple gel, # *P*< 0.05 in comparison to the chitosan-coated SPN-NLC gel

 The results demonstrated that the permeation of SPN-NLC gel through the epidermal layers was significantly higher compared to chitosan-coated SPN-NLC gel (*P*< 0.05). The receptor compartment analysis showed that the amount of SPN detected in the SPN-simple gel was greater (1559.547 ± 124.113 µg/cm^2^) than in the chitosan-coated SPN-NLC gel (342.142 ± 24.466 µg/cm^2^) and SPN-NLC gel (607.546 ± 82.890 µg/cm^2^) (Figure 5) (*P*< 0.05). Additionally, the average amount of SPN retained in the dermis was significantly lower for SPN-simple gel (332.297 ± 10.321 µg/cm^2^) compared to chitosan-coated SPN-NLC gel (2166.842 ± 240.279 µg/cm^2^) and SPN-NLC gel (1146.881 ± 204.679 µg/cm^2^) (*P*< 0.05) (Figure 6).

 The enhanced transdermal permeation of the lipid-based nanocarrier encapsulating SPN can be attributed to the specific characteristics of SPN and the barrier properties of the stratum corneum. One factor that contributes to the ability of SPN to penetrate the epidermis is its lipophilic nature. Additionally, the chemical composition of NLCs has been shown to significantly influence the permeation of SPN and its associated substances through the rat dermis. NLCs have been found to provide better occlusion and increased moisture to the stratum corneum, thereby impacting the transdermal penetration of active substances in the formulation.^43^

 The aim of a skin uptake assessment for SPN in acne treatment would typically be to evaluate the absorption and penetration of SPN into the skin when applied topically. By assessing the skin uptake of SPN, researchers can determine the drug’s ability to reach its target site in the skin and exert its intended therapeutic effects.

 Specifically, the assessment may involve studying factors such as the rate and extent of SPN absorption, the depth of penetration into the skin layers, and the concentration of the drug in various skin compartments. This information helps in understanding the pharmacokinetics and bioavailability of topically applied SPN.

 Additionally, the assessment may aim to compare the skin uptake of different formulations or delivery systems of SPN, such as gels, creams, or nanoparticle-based formulations. It can help determine which formulation optimally enhances drug penetration and ensures effective acne treatment. Also, skin uptake assessment was to be conducted for SPN nanoparticles in acne treatment, the aim would likely be to evaluate the ability of the nanoparticle formulation to enhance the delivery and penetration of SPN into the skin for acne treatment. Nanoparticle-based formulations can improve drug stability, increase drug solubility, and allow for controlled release and targeted delivery to the desired site.

 The assessment might involve investigating parameters such as the particle size, surface charge, and composition of the SPN nanoparticles to optimize skin penetration. Additionally, the assessment may evaluate the release kinetics of SPN from the nanoparticles, as well as the drug’s accumulation in the skin layers, to determine its effectiveness in treating acne lesions. Furthermore, the skin uptake assessment may compare the efficacy of SPN nanoparticles with other conventional formulations, such as creams or gels, to determine their relative advantages in terms of drug delivery and therapeutic outcomes.^3,44,45^

 Fluconazole and quercetin solid lipid nanoparticle (SLN) formulations have also demonstrated an increased localization of the substances in the skin.^20,46^ Research has shown that chitosan, as a biopolymer, can be used to control the release rate of substances in transdermal drug delivery systems. The bioadhesive properties of chitosan play a significant role in both dermal and transdermal applications. The stratum corneum, consisting of closely packed keratinocytes, has negative charges similar to epithelial cells. The interaction between the positive charges of chitosan and the negative charges of the epidermis contributes to its bioadhesive property, which can enhance percutaneous permeation.^47-52^

 Silva et al conducted a study on the cutaneous delivery of clobetasol propionate using NLC and chitosan-coated NLC formulations. The effectiveness of cutaneous delivery was confirmed through comprehensive in vitro cutaneous penetration investigations, along with the quantification of the pharmaceutical substance in various skin layers based on their physicochemical properties. The results showed that chitosan-coated NLC exhibited significantly higher drug accumulation in the cutaneous layer compared to uncoated NLC, with an increase of more than 80-fold. In contrast, the uncoated NLC formulation did not demonstrate any significant cutaneous accumulation.^53^

###  Properties of demography

**Figure 7:**
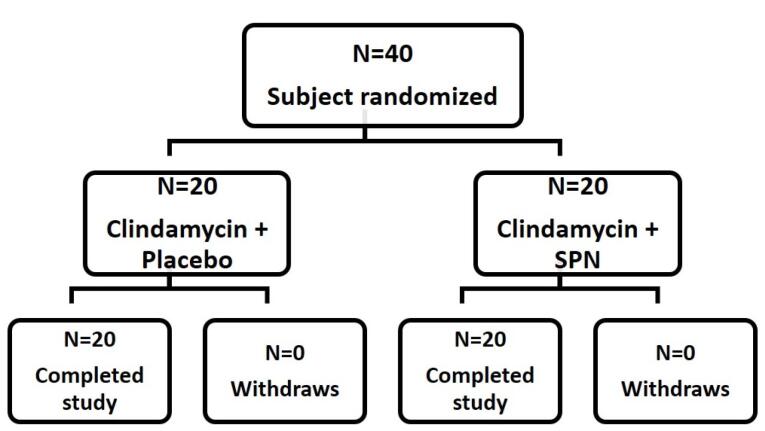
Profiles of a randomized trial

 In this study, a total of 40 participants with mild to moderate acne were studied. Among them, 20 participants (50%) were assigned to receive treatment with chitosan-coated SPN-NLC gel along with clindamycin 2% solution (treatment group), while the other 20 participants (50%) were assigned to receive treatment with chitosan-coated NLC gel without SPN along with clindamycin 2% solution (placebo group) (Figure 7). The participants in both groups were evaluated over an 8-week therapy period. The average age range in the treatment and placebo groups was 20.65 ± 3.884 and 19.65 ± 3.183 years, respectively. There were no significant differences in age between the subgroups (*P =*0.3788). The average ratings for inflammatory and non-inflammatory lesions were found to be comparable between the two groups.

###  Treatment effectiveness based on non-inflammatory and inflammatory lesion scores

**Figure 8:**
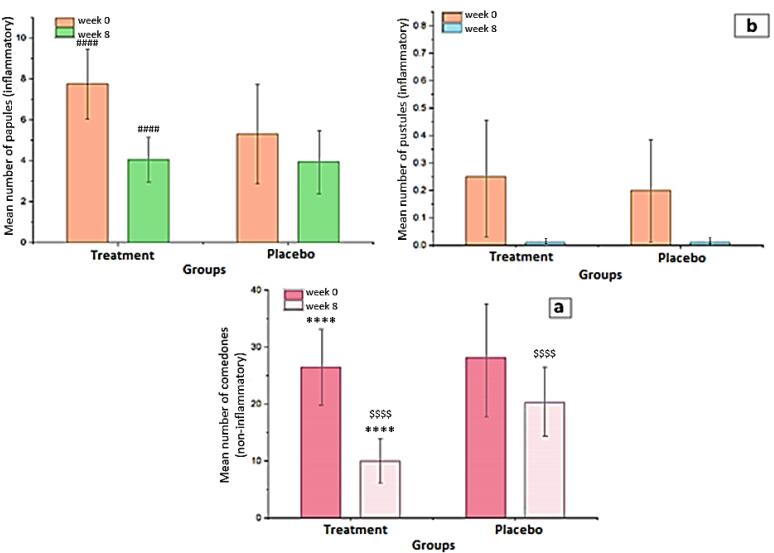
(a) An average number of the non-inflammatory lesions from baseline, and (b) An average number of the inflammatory lesions from baseline (mean ± SD). **** *P*< 0.0001 for intragroup analysis in the treatment group. #### *P =*0.0001 for intragroup analysis in the treatment group. $$$$ *P =*0.0001 for comparison between treatment and placebo groups in the 8th week

Figure 8a and 8b show the reductions in non-inflammatory and inflammatory lesions during the treatment period, respectively. While comparing week 8 to week 0, the mean count of non-inflammatory lesions in the treatment group was considerably fewer than in the placebo group (*P <*0.0001). The average count of the non-inflammatory lesion (comedones) declined in the treatment and placebo groups, from 26.45 ± 6.66 to 10.00 ± 3.9 and 28.15 ± 6.97 to 20.25 ± 6.29 respectively. The decrement of non-inflammatory lesions in the group receiving chitosan-coated SPN-NLCs was evaluated and found to be effective after 8 weeks of therapy with standard acne medicines. Figure 8b depicts the decline in inflammatory lesions through time. The number of pustules in the chitosan-coated SPN-NLC gel recipient group was 0.25 ± 0.44 at first, and 0.2 ± 0.523 in the control group. There is no considerable difference between the two groups, according to the evaluation (*P =*0.7464), and there is no significant difference between the groups at the end of 8 weeks of treatment due to complete healing. At the start of the research, the count of papules in the drug-receiving group was 7.75 ± 1.713 and 5.3 ± 2.430 in the control group. When the two groups are compared at the start of the study, the amount of inflammatory popular lesions in the drug group is higher than in the placebo group (*P =*0.0007). After 8 weeks of therapy, the number of papules in the drug-receiving group was 4.05 ± 1.099 and 3.95 ± 1.572 in the placebo group. After 8 weeks of treatment, there was no considerable difference between the two groups in terms of the count of inflammatory lesions (*P =*0.8169). The intragroup analysis also revealed that after eight weeks of therapy, the group receiving a topical product containing chitosan-coated SPN-NLCs gel had a greater reduction in lesions (*P =*0.0001) than the placebo group. Acne vulgaris dermatologic healing in the treatment group could be supported by a considerable decline in sebum production, which is excessive in acne and could contribute to the dermatologic healing of acne vulgaris in the treatment group.^54^ The 5% local SPN cream has shown effectiveness in acting as an anti-androgen in human sebaceous glands. It works by antagonizing dihydrotestosterone receptors, leading to a decrease in the levels of dihydrotestosterone, a hormone associated with sebum production and acne.^16^ According to the study conducted by Califano et al, the administration of 5% SPN cream resulted in complete resolution of acne in 30% of the participants, while 65% of the participants showed significant improvement and recovery from acne.^55^ Malek Afzali et al conducted a randomized placebo-controlled study to investigate the effectiveness of a 5% SPN gel in the treatment of facial acne.^16^ Kelidari et al conducted a randomized controlled trial to evaluate the efficacy of a 1% SPN-NLC gel compared to a 5% SPN gel in the treatment of facial acne.^7^

###  Effectiveness on TLC and ASI

**Figure 9:**
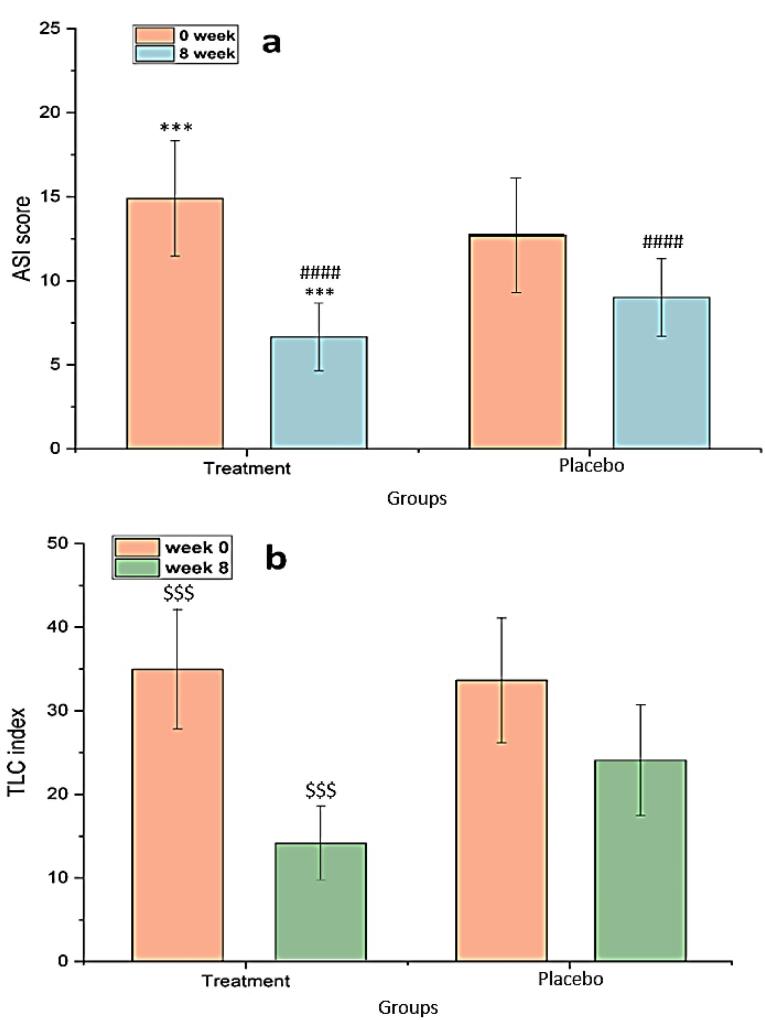
(a) ASI from baseline, and (b) TLC index from baseline (mean ± SD). *** *P*< 0.001 for intragroup ASI reduction analysis in the treatment group. #### *P*< 0.0001 for comparison of ASI between treatment and placebo groups in the 8th week. $$$ *P*< 0.001 for intragroup TLC reduction analysis in the treatment group

 In Figure 9a, a statistically significant difference was observed (comparing week 8 to week 0) in the treatment groups regarding a decrease in ASI (*P*< 0.001). The ASI in the treatment group decreased significantly from 14.9 ± 3.436 at the beginning to 6.562 ± 2.011 at the end of the trial (week 8). Comparing the two groups after 8 weeks of therapy revealed that the rate of ASI reduction in patients who received chitosan-coated SPN-NLC gel with conventional acne treatment was significantly higher than the group receiving placebo with conventional treatment (*P*< 0.0001). The TLC in the treatment and control groups was 34.95 ± 7.11 and 33.65 ± 7.45, respectively, at the start of the trial, and the index was significantly reduced to 14.05 ± 4.43 and 24.62 ± 6.61 after 8 weeks of therapy (Figure 9b). SPN-loaded chitosan-coated NLC gel showed a significant reduction in the number of lesions compared to single conventional acne treatment during the treatment period.

 The chitosan-coated NLC was selected due to the fact that the chitosan-coated formulations can enhance skin penetration of active ingredients compared to the uncoated formulation. Scalia et al investigated the enhancement of in vivo human skin penetration of resveratrol by chitosan-coated lipid microparticles.^56^ The investigation’s findings revealed that the cream with chitosan-coated lipid microparticles (LMs) was able to improve the penetration of resveratrol into the stratum corneum of humans, unlike the conventional uncoated LMs. The concentration of resveratrol in the horny layer has a significant impact, as it facilitates diffusion into the epidermis - the primary location for its efficacy.

 Also, the other benefit of chitosan usage is its antibacterial effect which can have a considerable effect on Acne treatment. Chitosan, a natural biopolymer, has significant potential for its antibacterial applications. With the implementation of appropriate techniques for nanoparticle synthesis, it is possible to create durable and highly effective chitosan nanoparticles for numerous industrial uses. The utilization of chitosan nanoparticles either independently or combined with other substances has demonstrated its inhibitory effects against both G− and G + bacteria.^57^

 Malek Afzali et al previously conducted a randomized placebo-controlled trial to assess the effectiveness of a 5% SPN gel in the treatment of facial acne.^16^ In their study, Malek Afzali et al observed significant reductions in TLC and ASI in both the placebo and SPN groups. They suggested that the alcoholic concentration of the gel may have contributed to these improvements in both groups. Similar findings were observed in the placebo group, indicating a potential impact of the gel formulation itself. However, the chitosan-coated SPN-NLC gel may have provided additional benefits by targeting specific cutaneous structures, such as pilosebaceous structures including hair follicles and sebaceous glands. This alternative pathway for NLC cutaneous penetration could result in greater uptake of smaller molecules during the post-administration period. Additionally, Kelidari et al demonstrated that SPN-NLC significantly reduced the TLC index during acne therapy, further supporting the potential efficacy of NLC-based formulations.^7^

 In a study conducted by Walton et al, local administration of SPN at concentrations of 3% and 5% did not have any impact on sebum secretion in individuals. The researchers hypothesized that the lack of an anti-androgen effect from the local SPN cream may be attributed to the carrier used in the formulation. They suggested that the carrier needed to be effective in delivering the medication to the specific sebaceous glands in order to exert its anti-androgenic effects.^58^ Pilosebaceous units are being studied for their potential use as depots for local treatment as well as a transfer mechanism for systemic medication delivery. Furthermore, the hair follicles itself is the specific target for various hair follicle-associated disorders, including acne.^59^ Certain researchers propose that the presence of a lipid coating or lipophilic properties of substances may enhance their absorption into hair follicles. This is because hair follicles are rich in sebum, which provides a lipophilic environment. The sebum present in hair follicles creates a favorable milieu for the penetration and accumulation of lipophilic substances, potentially facilitating their targeted delivery and efficacy in treating hair follicle-related conditions.^60^ The pilosebaceous unit, which includes the hair follicle and associated sebaceous glands, has been recognized as a highly absorptive structure compared to the outermost layer of the skin (corneocytes). This makes follicular delivery a significant pathway for the in vivo uptake of locally administered drugs. Studies have shown that particle sizes larger than 10 µm tend to remain on the skin surface, while particles ranging from 3 to 10 µm can concentrate within the hair follicles. Particles smaller than 3 µm can penetrate and enter the follicles, potentially facilitating targeted drug delivery to this specific area of the skin.^61^ Indeed, the particle diameter of the chitosan-coated SPN-NLC used in this study (408 nm) suggests that it has the potential to enter the hair follicles and contribute to the reduction of lesions. Compared to conventional formulations, the lipid components present in NLC can have an impact on the interactions with the skin’s dermal layer. Lipid-based vehicles can adhere to the skin’s surface, leading to localized interactions with the superficial junctions between corneocyte structures and the pathways between corneocyte cells. This, in turn, can facilitate drug permeability by reducing the compactness of corneocytes and increasing the size of intercorneocyte gaps. These effects of lipid-based particles may enhance the penetration and distribution of SPN in the skin, particularly within the hair follicles, thereby potentially improving the therapeutic outcomes for treating skin lesions.^62^

###  Patient compliance and side effects of products

**Figure 10:**
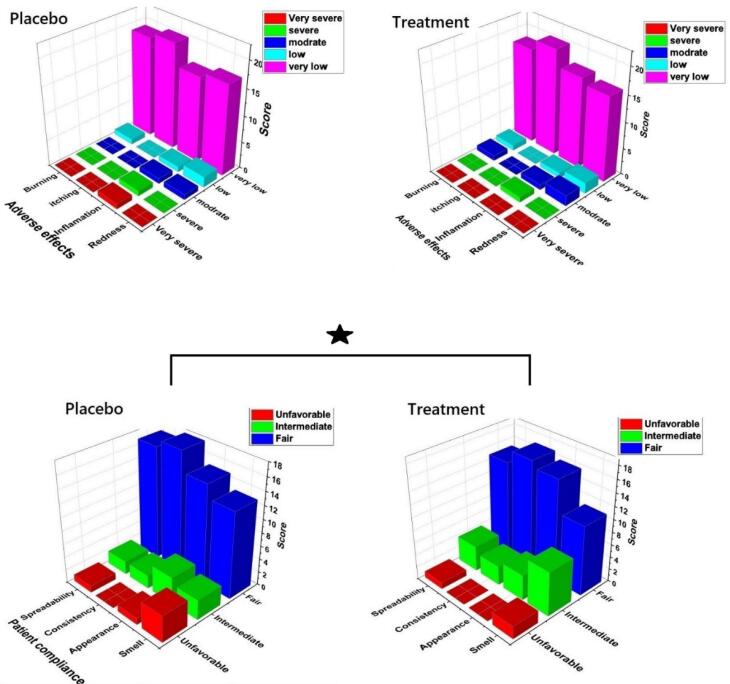
(a) The adverse effect of product and placebo on patients, (*P* > 0.05) (b) Patients’ compliance after application of the product and placebo (*P* < 0.05)

Figure 10 presents the data regarding the participants’ skin conditions in both groups. The results indicate that there were no significant differences between the two groups in terms of redness, inflammation, itching, and burning sensations (Figure 10a). This suggests that the use of the chitosan-coated SPN-NLC gel did not lead to notable changes or adverse reactions compared to the placebo group. Additionally, the feedback from the patients regarding the product’s characteristics, such as its smell, appearance, spreadability, and consistency, showed high levels of satisfaction (Figure 10b).

 The importance lies in ensuring that pharmaceutical substance delivery platforms not only effectively release the desired substance but also avoid causing significant irritation, redness, and inflammation. Balancing both objectives is crucial for the success of such platforms as it allows for effective delivery without adverse effects on the skin.^25^

 In topical formulations, skin irritation is a common adverse effect that is often associated with the quantity of the pharmaceutical substance present in the formulation. To mitigate this adverse effect, it is important to regulate the release of the pharmaceutical substance from the formulation. By enhancing intra-follicular penetration and reducing skin absorption, the adverse effects of the formulation can be effectively controlled.^13^ Previous research has advocated for the use of lipidic nanoparticles as promising vehicles to enhance the therapeutic efficacy of local medications and mitigate adverse effects.^62^

## Conclusion

 SPN was successfully encapsulated within chitosan-coated NLC, which was prepared using a combination of nonionic surfactants with varying HLB numbers and palmitic acid. Solid-phase studies confirmed that SPN maintained its amorphous form in the formulation without any chemical interactions with other components of the chitosan-coated SPN-NLC. The optimized NLC formulation exhibited a small particle size with a narrow size distribution, and the EE was approximately 82%. The results of this randomized double-blind trial confirmed that the treatment with chitosan-coated SPN-NLC was well-tolerated and led to significantly improved healing of mild to moderate acne vulgaris after 8 weeks of therapy compared to the beginning condition. The formulated gel effectively treated both non-inflammatory and inflammatory lesions without causing any adverse effects on the skin. However, further clinical evaluation is necessary to assess the efficacy of the chitosan-coated SPN-NLC gel in a larger sample size and across different locations.

## Acknowledgments

 Current study supported by an award from the research council of Mazandaran University of Medical Sciences, Sari, Iran.

## Competing Interests

 No potential conflict of interest was reported by the authors.

## Ethical Approval

 All animal investigations were authorized by the MAZUMS’ Ethics Review Board for Animal Research under registration code≠IR.MAZUMS.REC.1399.691. The clinical trial procedures were carried out according to the guidelines of the Ethics Committee of Mazandaran University of Medical Sciences (IRCT20120707010203N11).

## Funding

 This study was funded by the Mazandaran University of Medical Sciences Research Council, Sari, Iran.
